# Paralysis after Diphtheria

**Published:** 1890-05-03

**Authors:** 


### PARALYSIS AFTER DIPHTHERIA.

Dr. David Hausemann contributes to "Virchow's Archives" an account of the unusually extensive nervous phenomena that followed an attack of diphtheria contracted by himself in the performance of his duties as resident medical officer of a hospital. After an attack of scarlatina he remained for two years very susceptible to infection of various kinds as indicated by frequent sore throats, &c. He had much to do with post-mortem examinations of diphtheritic cases, and at length took the disease himself, though apparently not in a severe form. Albuminuria, however, persisted for a full month. On the eighteenth day he noticed a slight difficulty in swallowing, but his general health improved and his strength increased, the pulse, though accelerated, being regular and fairly strong. But about six weeks from the commencement of his illness he had an attack of syncope, which was followed by paræsthesiæ everywhere except in the palate, fauces, pharynx, and larynx. To these succeeded anæsthesiæ and pareses, the head being most affected and the trunk and upper limbs more than the lower. Taking the nerves in order: I., cranial (olfactory) was paralyzed; II., intact; III., ditto, except as regards the reaction of the pupils; IV., ditto; V., paralyzed, especially in the second branch; VI., slightly paralyzed; VII., paralyzed throughout the pes anserina, except in the superior cutaneous branch of the neck; VIII., intact; IX., paralyzed in the pharyngeal and tonsillary branches; X., in those of the heart, larynx, and œsophagus; XI., in the muscular laryngeal and pharyngeal communications; and XII., in those of the tongue. In the extremities he suffered from motor and sensory disturbances, ataxy, and slight atrophy of the muscles, with hyperalgesia in the upper limbs. In the abdomen there was slight parasthesia, though towards the waist sensation was normal, as were the functions of the bladder and rectum, except that the latter were performed without sensation. When the symptoms dependent on the paralyses of the cranial nerves improved those of the extremities and abdomen became worse. Movements resembling athetosis appeared in the hands, uncontrolled by the aid of sight. In the course of three or four months, with good diet, iron, and exercise in the open air, a decided recovery took place. He notices—(1) The extraordinary extension of the ataxia, including under this term the inability to produce tones of any given pitch or character; (2) that recovery from the primary disease was almost complete when these phenomena began to appear; (3) the double path or course of these disturbances; (*a*) through the vascular and lymphatic systems, as shown by nephritis, both parenchymatous and interstitial and myocarditis; and (*b*) through the nervous system both centripetal and centrifugal. Such an experience as Dr. Hausemann's is probably unique, though some or other of these phenomena variously associated are common enough.

